# Unveiling the efficacy of *Bacillus faecalis* and composted biochar in alleviating arsenic toxicity in maize

**DOI:** 10.1186/s12870-024-05372-2

**Published:** 2024-07-11

**Authors:** Yonghui Liao, Humaira Ashraf, Shoucheng Huang, Musarrat Ramzan, Rabia Saba, Muhammad Baqir, Saleh H. Salmen, Sulaiman Ali Alharbi, Misbah Hareem

**Affiliations:** 1 School of Life Science, Jinggangshan University, Ji’an, 343009 Jiangxi China; 2 Department of Botany, Faculty of Chemical and Biological Sciences, The Islamia University of Bahawalpur, Bahawalpur, Punjab Pakistan; 3 College of Life and Health Science, Anhui Science and Technology University, Fengyang, 233100 Anhui China; 4 Department of Botany, University of Thal Bhakkar, Bhakkar, Punjab Pakistan; 5 Department of Soil and Environmental Sciences, MNS University of Agriculture, Multan, Punjab Pakistan; 6 Department of Botany and Microbiology, College of Science, King Saud University, PO Box -2455, Riyadh, 11451 Saudi Arabia; 7 Department of Environmental Sciences, Woman University Multan, Multan, Punjab Pakistan

**Keywords:** Chlorophyll contents, Growth attributes, Heavy metal, Organic amendment, Rhizobacteria, *Zea mays* L

## Abstract

Arsenic (As) contamination is a major environmental pollutant that adversely affects plant physiological processes and can hinder nutrients and water availability. Such conditions ultimately resulted in stunted growth, low yield, and poor plant health. Using rhizobacteria and composted biochar (ECB) can effectively overcome this problem. Rhizobacteria have the potential to enhance plant growth by promoting nutrient uptake, producing growth hormones, and suppressing diseases. Composted biochar can enhance plant growth by improving aeration, water retention, and nutrient cycling. Its porous structure supports beneficial microorganisms, increasing nutrient uptake and resilience to stressors, ultimately boosting yields while sequestering carbon. Therefore, the current study was conducted to investigate the combined effect of previously isolated *Bacillus faecalis* (*B. faecalis*) and ECB as amendments on maize cultivated under different As levels (0, 300, 600 mg As/kg soil). Four treatments (control, 0.5% composted biochar (0.5ECB), *B. faecalis*, and 0.5ECB + *B. faecalis*) were applied in four replications following a completely randomized design. Results showed that the 0.5ECB + *B. faecalis* treatment led to a significant rise in maize plant height (~ 99%), shoot length (~ 55%), root length (~ 82%), shoot fresh (~ 87%), and shoot dry weight (~ 96%), root fresh (~ 97%), and dry weight (~ 91%) over the control under 600As stress. There was a notable increase in maize chlorophyll a (~ 99%), chlorophyll b (~ 81%), total chlorophyll (~ 94%), and shoot N, P, and K concentration compared to control under As stress, also showing the potential of 0.5ECB + *B. faecalis* treatment. Consequently, the findings suggest that applying 0.5ECB + *B. faecalis* is a strategy for alleviating As stress in maize plants.

## Introduction

Among different heavy metals [1–6], arsenic (As) can severely impact plant growth and development due to its toxicity [7]. When plants absorb As, it disrupts various physiological processes, leading to stunted growth, reduced biomass accumulation, and impaired nutrient uptake [8]. Furthermore, As also has detrimental effects on plants, affecting photosynthesis, enzyme activities, and cellular functions [9]. It induces oxidative stress, generating harmful reactive oxygen species (ROS) that damage cell components [10]. Such conditions severely hamper plant growth and productivity, especially in contaminated areas. The need for time is to adopt effective mitigation measures to preserve crop health and yield.

Rhizobacteria, also known as plant growth-promoting rhizobacteria (PGPR), are important for enhancing plant health through various growth-promoting mechanisms [11]. They thrive on root exudates, which contain essential nutrients like free amino acids, carbohydrates, vitamins, and crucial elements, providing them with the necessary nourishment [12]. They also contribute significantly to plant growth and development by synthesizing phytohormones that stimulate growth, fixing atmospheric nitrogen (N), and producing enzymes that aid in mineral solubilization, thus regulating plant growth [13, 14].

Composted biochar (ECB) is a combination of organic materials (compost) and pyrolyzed waste materials [15–17]. It can support the activity of rhizobacteria and helps counteract heavy metal toxicity in soil by creating a favorable environment for plant growth [18–22]. Its porous structure offers a protected habitat for beneficial rhizobacteria, fostering their interaction with plant roots and reducing arsenic availability [23, 24]. This dual effect protects plants from arsenic toxicity while enhancing soil structure and nutrient retention capacity [25], showing composted biochar’s potential to boost plant-microbe partnerships and alleviate environmental pressures.

Maize, a vital crop in global agriculture from the *Poaceae* family, plays diverse roles in food production, providing essentials like grits, chapatti, and starch solutions [26]. Rich in phytochemicals such as proteins, vitamins, minerals, ashes, and amino acids, maize contributes to human well-being [27]. Its high carbohydrate content makes it an excellent energy source. However, arsenic toxicity threatens maize cultivation by hindering water and nutrient absorption, leading to stunted growth and lower yields [22, 28, 29].

A current study was conducted to investigate the influence of *Bacillus faecalis*(*B. faecalis*) rhizobacteria and ECB on maize cultivated with and without As toxicity. This research covers the knowledge gap regarding the use of *B. faecalis* and ECB as sole and combined amendments against As toxicity in maize. The novelty of the current study lies in the selection of *B. faecalis* and ECB as sole or combined efficacious amendments for the minimization of As toxicity in maize. It is hypothesized that *B. faecalis* with ECB might have better potential to improve maize growth with and without As toxicity.

## Materials and methods

### Pot experiment setup

A pot experiment was conducted in the research area of the Department of Botany Islamia University Bahawalpur to examine the effect of *Bacillus faecalis* inoculation and composted biochar on the growth, nutrient concentration, and antioxidant activity in maize cultivated in arsenic (As) contaminated soil. A composite soil sample was made using six samples for the pre-experimental soil analysis. The pre-experimental soil characteristics are provided in Table 1.

**Table 1:** Pre-experimental soil and irrigation characteristics

**Soil**	**Values**	**References**
pH	8.45	[30]
EC*e* (dS/m)	3.34	[31]
SOM (%)	0.40	[32]
TN (%)	0.0025	[33]
AP (µg/g)	5.79	[34]
EK (µg/g)	111	[35]
ENa (µg/g)	144	[36]
Texture	Clay Loam	[37]
**Irrigation**	**Values**	**References**
pH	7.02	[38]
EC (µS/cm)	467	
Carbonates (meq./L)	0.00	
Bicarbonates (meq./L)	5.29	
Chloride (meq./L)	0.01	
Ca + Mg (meq./L)	3.19	
Sodium (mg/L)	101	

TN = Total Nitrogen; AP = Available Phosphorus; EK = Extractable Potassium; ENa = Extractable Sodium

*References are showing methods used for the characterization of soil and irrigation water

### Biochar Preparation

Citrus fruit waste was sourced from a local fruit and vegetable market, situated at coordinates 30°11’29.8’’N and 71°28’48.8’’E, and was used to produce biochar. The collected waste was initially sun-dried and subsequently cut into small pieces. These prepared waste materials underwent pyrolysis under aerobic conditions at a controlled temperature of 525 ± 11 °C. Compost and biochar were mixed manually in a 1:1 ratio [15] following incubation for 21 days to get a homogenized product. The physicochemical properties of the composted biochar generated in the pre-experimental phase are summarized in Table 2.

**Table 2:** Pre-experimental biochar and compost characteristics

Biochar	Values		Compost	Values		B. faecalis	Characteristics
pH*s*	7.98	[30]	pH*s*	6.66	[30]	Accession number	MW475277
EC*e* (dS/m)	3.89	[31]	EC*e* (dS/m)	5.21	[31]	ACC deaminase activity	+ve
Ash Content (%)	32	[39]	TP (%)	0.88	[40, 41]	IAA Production	-ve
Volatile Matter (%)	22		TN (%)	1.52		Zn Solubilization	+ve
Fixed carbon (%)	46		TK (%)	0.48		Siderophore production	+ve
TP (%)	0.29	[40, 41]	TN = Total Nitrogen EP = Extractable Phosphorus AK = Available Potassium CEC = Cation Exchange Capacity EC = Electrical Conductivity			Chitinase production	+ve
TN (%)	0.37					HCN production	-ve
TK (%)	0.15					Catalase + Oxidase	+ve

* Plus sign (+) shows the presence, while a minus sign (-) indicates the absence of the functions. References are showing methods used for the characterization of biochar and compost

### Seed collection and sterilization

In this study, maize seeds (Cimmyt-Pak) were purchased from a seed dealer of the Government of Punjab in Multan, Punjab, Pakistan. A two-step process was employed to ensure surface sterilization. First, the seeds were immersed in 70% ethanol for 5 min, followed by a 10-minute treatment with 5% sodium hypochlorite. Subsequently, the seeds were thoroughly rinsed with distilled water and left to soak for 24 h.

### Characterization for genetic and biochemical traits

The isolate was cultured on LB agar plates and sent to Macrogen in South Korea for 16 S rRNA sequencing through Sanger technology. PCR amplification was performed using the primers 27 F (5’-AGA GTT TGA TCM TGG CTC AG-3’) and 1492R (5’-TAC GGY TAC CTT GTT ACG ACT T-3’), along with the universal sequencing primers 785 F (5’-GGA TTA GAT ACC CTG GTA-3’) and 907R (5’-CCG TCA ATT CMT TTR AGT TT-3’) to ensure overlapping sequences. The resulting crude sequences were then trimmed and edited using BioEdit software [42]. The final sequences were performed [43] to compare a query sequence against a public sequence to find the closest related organism in the Genbank database. Enzyme 1-aminocyclopropane-1- carboxylate (ACC) deaminase activity was tested using the method of Glick et al. [44]. As described by Sarwar et al. [45], the protocol was followed to determine Indole-3-acetic acid (IAA) production [45]. Isolate’s ability to solubilize zinc was detected following the procedure, as Kumar et al. reported [46]. Siderophore production was tested following the method described by Schwyn and Neilands [47]. A qualitative test was performed to determine chitinase production following the technique of Dunne et al. [48]. HCN (cyanogen) production was tested using the procedure described by Bakker and Schippers [49].

### Inoculum preparation and seed inoculation

The culture of *B. faecalis* was prepared in an erlenmeyer flask (50 ml) containing 20 ml of Luria-Bertani (LB) broth media (composition per liter; 10 g tryptone, 5 g yeast extract, 10 g NaCl, and 7.2 pH) [50]. The flasks were incubated at 28 ± 1 °C for 72 h in a shaking incubator (100 rpm). After that, broth culture was shaken at 4 °C for 15 min to harvest the bacterial cells. Using spectrophotometer, inoculum optical density was maintained 0.5 in sterile LB broth media.

Inoculation of maize seeds with *B. faecalis* was performed using peat and 10% sugar solution as a sticky material. For 50 g seeds, 10 g peat was used while 10 ml of sugar solution was added. After inoculation, the seeds were allowed to dry under controlled conditions to ensure proper adhesion of the inoculum to the seed surface. The plant growth-promoting characteristics of *B. faecalis* are provided in Table 2.

### Treatment plan

Total 4 treatments, i.e., control (no *B. faecalis* + no 0.5ECB), *B. faecalis*, 0.5% composted biochar (0.5ECB) and *B. faecalis* + 0.50ECB were applied under 0 (0As), 300 (300As) and 600 mg As/ kg soil (600 As) following completely randomized design. The levels of As were selected on the basis of literature [51, 52]. For the introduction of As toxicity as per the treatment plan, As_2_O_3_ was used. The salt was Sigma-Aldrich’s product, which had CAS No.=1327-53-3; Batch No.=BCCL3474; faint grey in colour and powder form. All treatments were applied in 4 replicates [48, 49]. For the artificial spiking of As at the rate of 300 and 600 mg As/ kg soil As_2_O_3_ was mixed manually. After that, 65% moisture content was maintained in the soil, and it was incubated at 25 ± 3 °C for 21 days. The mixing was continued during the incubation period with an interval of 7 days.

### Pot preparation, sowing and growth conditions

A plastic container with dimensions of 15 inches in width and 45 inches in depth was loaded with 10 kg of soil. The initial physicochemical characteristics of the soil before the experiment are outlined in Table 1. Ten seeds were sown in each container, and, following a period of seven days from germination, two healthy seedlings were retained after thinning. The light (500 µmol m^− 2^ s^− 1^) was maintained 16-hour light/8-hour dark cycle using a combination of natural sunlight supplemented with LED bulb. Throughout the experiment, temperature was maintained at 25 ± 10 °C while humidity was 55 ± 7%.

### Fertilizer

To address the maize nutritional requirements, the soil was enriched with nitrogen (N), phosphorus (P), and potassium (K) in recommended amounts: 294 kg per ha of nitrogen (~ 1.46 g per 10 kg of soil), 170 kg per ha of phosphorus (~ 0.85 g per 10 kg of soil), and 124 kg per ha of potassium (~ 0.62 g per 10 kg of soil). The fertilizers were mixed in soil at the time of pot preparation. Urea served as the nitrogen source, while single superphosphate was administered for phosphorus and potassium, meeting the prescribed criteria.

### Irrigation

At the beginning of the experiment, 100 ml of sterilized water was used for the initial irrigation of each pot. Subsequently, a daily water supply of 50 ml was given to each pot until the seedlings were ready for harvesting. The initial 100 ml water was added to ensure that the soil in each pot maintained a field capacity of 60%.

### Harvesting and data collection

After 35 days from the sowing date, the seedlings were harvested. Various morphological attributes, including shoot and root length and fresh and dry weights of shoot, leaves, and root, were measured immediately after harvesting using a standard measuring scale and an analytical grade digital balance. For the determination of dry weight, samples were oven-dried at 70 °C for 72 h to achieve the constant weight. Additionally, fresh leaf samples were collected and stored in liquid nitrogen to preserve them for further biochemical analysis.

### Chlorophyll contents

A pestle and mortar were used to crush 0.5 g of freshly obtained leaf samples with 20 ml of 80% acetone to measure the chlorophyll content. The mixture was centrifuged for 15 min at 3000 rpm rotations per minute, and absorbance readings were taken by taking 1 ml supernatant in a glass cuvette at wavelengths of 645 and 663 nm using a spectrophotometer (UV-1280 UV-VIS Spectrophotometer, Shimadzu) [53].

$$\text{Chlorophyll}\, \text{a} \left(\frac{\text{mg}}{\text{g}}\right)=\frac{\left(12.7 \times \text{A}663\right)- \left(2.69 \times \text{A}645\right)\times \text{V}}{1000 \times \text{W}}$$

$$\text{Chlorophyll}\, \text{b} \left(\frac{\text{mg}}{\text{g}}\right)=\frac{\left(22.9 \times \text{A}645\right)- \left(4.68 \times \text{A}663\right)\times \text{V}}{1000 \times \text{W}}$$

$$\text{Total}\, \text{Chlorophyll} \left(\frac{\text{mg}}{\text{g}}\right)=\frac{20.2\left(\text{A}645\right)+8.02\left(\text{A}663\right)\times \text{V}}{1000 \times \text{W}}$$

### Measurement of antioxidant activity

We assessed superoxide dismutase (SOD) activity on a spectrophotometer (UV-1280 UV-VIS Spectrophotometer, Shimadzu) by observing the inhibition of nitro blue tetrazolium (NBT) reduction in the presence of riboflavin [54]. Peroxidase (POD) activity was determined using a spectrophotometer (UV-1280 UV-VIS Spectrophotometer, Shimadzu) following the protocol outlined by [55]. Catalase (CAT) activity was evaluated by measuring the decrease in absorbance at 240 nm on a spectrophotometer (UV-1280 UV-VIS Spectrophotometer, Shimadzu) resulting from H_2_O_2_ decomposition [56]. Ascorbate peroxidase (APX) activity was determined by monitoring ascorbate oxidation in the presence of hydrogen peroxide (H_2_O_2_) [57]. To measure malondialdehyde (MDA) levels, we exposed sample extracts to a reaction with thiobarbituric acid (TBA) to form a coloured complex, then measured absorbance at 532 nm using a spectrophotometer (UV-1280 UV-VIS Spectrophotometer, Shimadzu) [58]. The assessment of Glutathione reductase (GR) activity involved observing the rate of NADPH oxidation using the extinction coefficient of NADPH, measured by monitoring the decrease in absorbance at 340 nm using a spectrophotometer (UV-1280 UV-VIS Spectrophotometer, Shimadzu) over one minute [59].

### Electrolyte Leakage

Initially, leaves were cleaned with deionized water to examine electrolyte leakage. We then placed standardized leaf samples in test tubes filled with 20 ml of deionized water and kept them at 25 °C for 24 h. Afterwards, we used a pre-calibrated EC meter (Jenway, 3540 pH-Conductivity Meter) to measure the electrical conductivity of the water solution. A second measurement was taken after subjecting the samples to a 20-minute heat treatment [60].

$$\text{Electrolyte}\, \text{Leakage} \left(\text{\%}\right)=\left(\frac{\text{EC}1}{\text{EC}2}\right)\times 100$$

### Relative water content

The study used a standard procedure [61] to determine the relative water content (RWC) of freshly harvested leaves. Leaf samples were collected, weighed, and immersed in distilled water until full turgidity. After drying, the final dry weight was determined using Ohaus PA 214 Pioneer Series digital balance. The RWC was calculated using a formula.

$$\text{RWC} \left(\text{\%}\right)=\left({\text{FW}}-{\text{DW}}\right)/\left({\text{TW}}-{\text{DW}}\right) \times 100$$

### Leaves and root nutrients concentration

Initially, roots and leaves samples were digested by following the standard protocol [62]. For the analysis of total nitrogen in samples, a glass-made manual Kjeldahl distillation apparatus was used [62]. Phosphorus was analyzed using a yellow colour method using ammonium molybdate and ammonium metavanadate. The final absorbance was taken at 420 nm wavelength using a spectrophotometer (UV-1280 UV-VIS Spectrophotometer, Shimadzu). For the assessment of potassium, samples were run on flame-photometer (Microprocessor Flame Photometer (VSI-604)) [36].

### Statistical analysis

The data was subjected to conventional statistical analysis [63]. OriginPro software was used to do a two-way ANOVA. Tukey’s test was applied for the comparison of treatment significance at *p* ≤ 0.05 [64].

## Results

### Shoot length

**Fig. 1:**
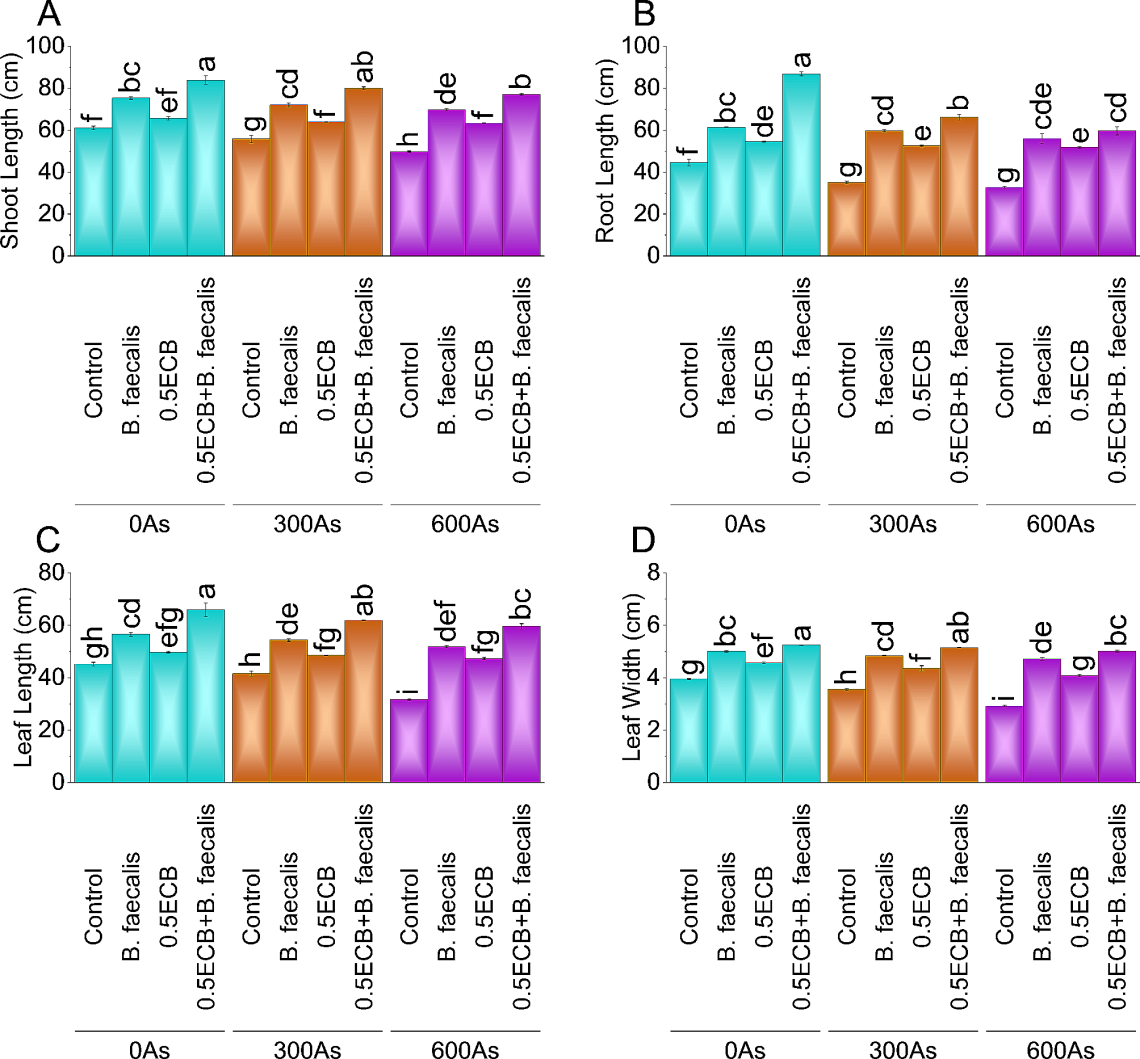
Influence of *B. faecalis* and composted biochar sole and combined application on maize shoot length (**A**), root length (**B**), leaf length (**C**), and leaf width (**D**) under 0 (0As), 300 (300As) and 600 mg As/kg soil (600As). Bars are an average of four replicates ± SE compared using Tukey’s test. Different letters showed significant changes at *p* ≤ 0.05

Compared to the control, *B. faecalis* application alone increased shoot length by ~ 23%, ~ 29%, and ~ 40% at 0As, 300As, and 600As stress conditions, respectively. The application of 0.5ECB also impacted shoot length, with increases of ~ 8%, ~ 14%, and ~ 28% at 0As, 300As, and 600As stress conditions, respectively. Notably the applications of 0.5ECB + *B. faecalis* exhibited the highest percentage increases in shoot length at ~ 37%, ~ 43%, and ~ 55% for the respective 0As, 300As, and 600As stress conditions compared to the control (Fig. 1A).

### Root length

Compared to the control, *B. faecalis* alone significantly increased root length by ~ 38%, ~ 70%, and ~ 71% under 0As, 300As, and 600As stress conditions. Similarly, the application of 0.5ECB alone positively impacted root length, with increases of ~ 22%, ~ 50%, and ~ 58% under the stress conditions. Notably, 0.5ECB + *B. faecalis* exhibited the highest percentage increase in root length, recording values of ~ 95%, ~ 88%, and ~ 82% for the 0As, 300As, and 600As stress conditions, respectively, compared to the control (Fig. 1B).

### Leaf length

Under 0As stress conditions, applying *B. faecalis* increased leaf length by ~ 25% and 0.5ECB by ~ 10%, while 0.5ECB + *B. faecalis* showed a remarkable ~ 46% increase compared to the control. In 300As stress, the application of *B. faecalis* increased leaf length by ~ 31%, 0.5ECB by ~ 17%, and 0.5ECB + *B. faecalis* by ~ 49%, respectively, over the control. In the 600As stress condition, leaf length increased by ~ 64% with *B. faecalis*, ~ 50% with 0.5ECB, and 0.5ECB + *B. faecalis* applications, leading to an ~ 88% increase over the control (Fig. 1C).

### Leaf width

Compared to the control group under 0As stress, the application of *B. faecalis* increased leaf width by ~ 27%, 0.5ECB by ~ 15%, and the application of 0.5ECB + *B. faecalis* by ~ 33%. In the 300As stress condition, *B. faecalis* increased leaf width by ~ 36%, 0.5ECB by ~ 23%, and 0.5ECB + *B. faecalis* by ~ 45% over the control. Under 600As stress, *B. faecalis*, 0.5ECB, and 0.5ECB + *B. faecalis* increased leaf width by ~ 62%, ~ 40%, and ~ 72%, respectively, compared to the control (Fig. 1D).

### Plant height

**Fig. 2:**
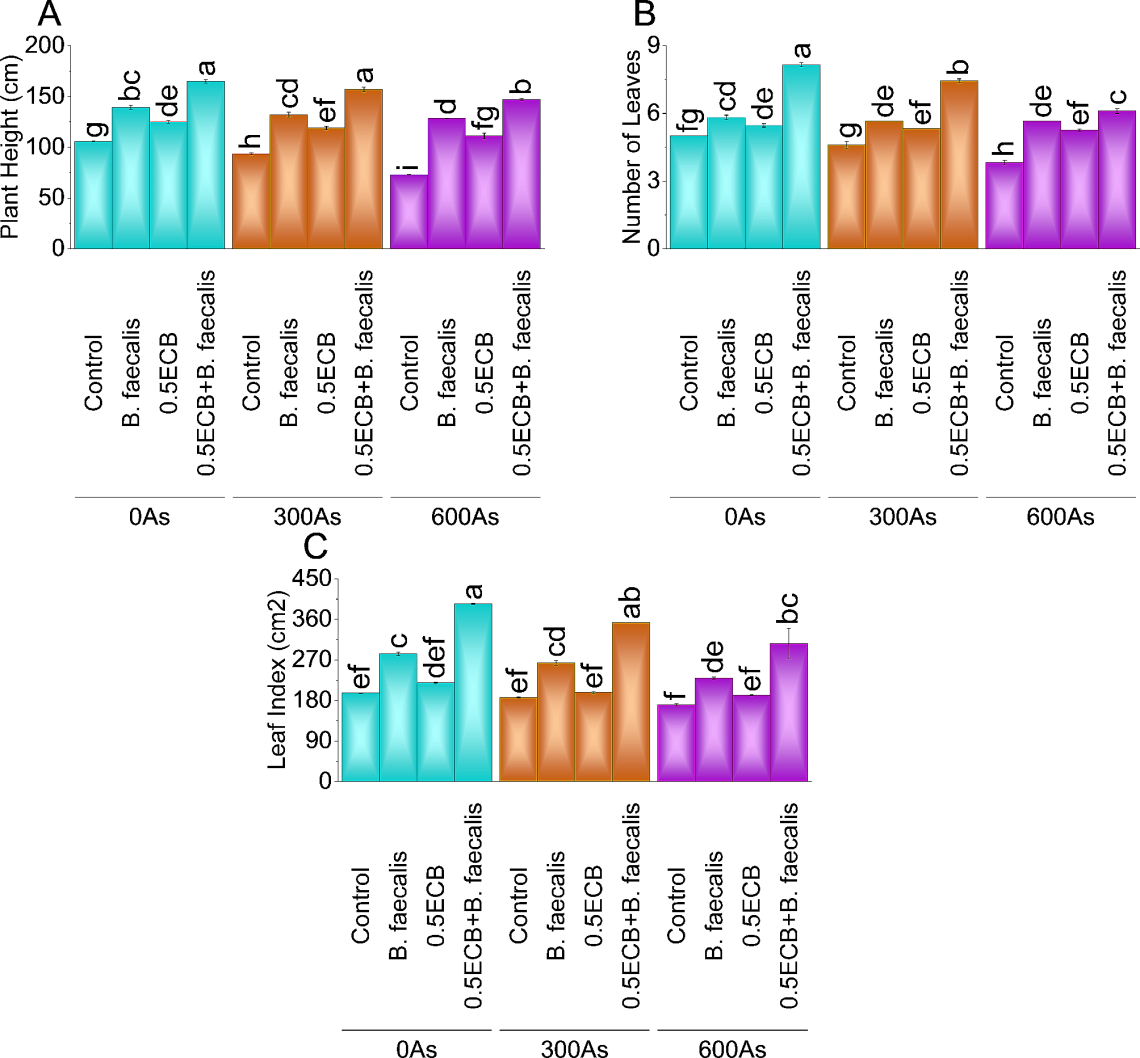
Influence of *B. faecalis* and composted biochar sole and combined application on maize plant height (**A**), number of leaves (**B**), and leaf index (**C**) under 0 (0As), 300 (300As) and 600 mg As/kg soil (600As). Bars are an average of four replicates ± SE compared using Tukey’s test. Different letters showed significant changes at *p* ≤ 0.05

In the 0As stress environment, applying *B. faecalis* and 0.5ECB individually resulted in a ~ 32% and ~ 18% increase in plant height, respectively. Notably, the combined application of 0.5ECB + *B. faecalis* led to a substantial ~ 56% elevation compared to the control. Similarly, under 300As stress, *B. faecalis* + 0.5ECB showed a ~ 41% and ~ 27% increase, while the 0.5ECB + *B. faecalis* application exhibited a significant ~ 68% rise over the control. Under 600As stress, the applications of *B. faecalis*, 0.5ECB, and 0.5ECB + *B. faecalis* increased plant height by ~ 76%, ~ 52%, and ~ 99%, respectively, compared to the control (Fig. 2A).

### Number of leaves

Under 0As stress, *B. faecalis* increased the number of leaves by ~ 17%, 0.5ECB by ~ 10%, and 0.5ECB + *B. faecalis* by ~ 63% compared to the control. In the 300As stress condition, *B. faecalis* increased the number of leaves by ~ 23%, 0.5ECB by ~ 16%, and 0.5ECB + *B. faecalis* by ~ 62% over the control. For 600As, *B. faecalis* increased by ~ 48%, 0.5ECB by ~ 37%, and 0.5ECB + *B. faecalis* by ~ 60% compared to control (Fig. 2B).

### Leaf index

In the 0As stress, *B. faecalis* increased leaf index by ~ 44% and 0.5ECB by ~ 12%. The application of 0.5ECB + *B. faecalis* showed a notable ~ 99% elevation over control. For the 300As stress condition, *B. faecalis* increased leaf index by ~ 41%, 0.5ECB by ~ 6%, and 0.5ECB + *B. faecalis* by ~ 89% over control. Under 600As stress, *B. faecalis* rose by ~ 35%, 0.5ECB by ~ 12%, and 0.5ECB + *B. faecalis* by ~ 80% compared to control (Fig. 2C).

### Shoot fresh weight

**Fig. 3:**
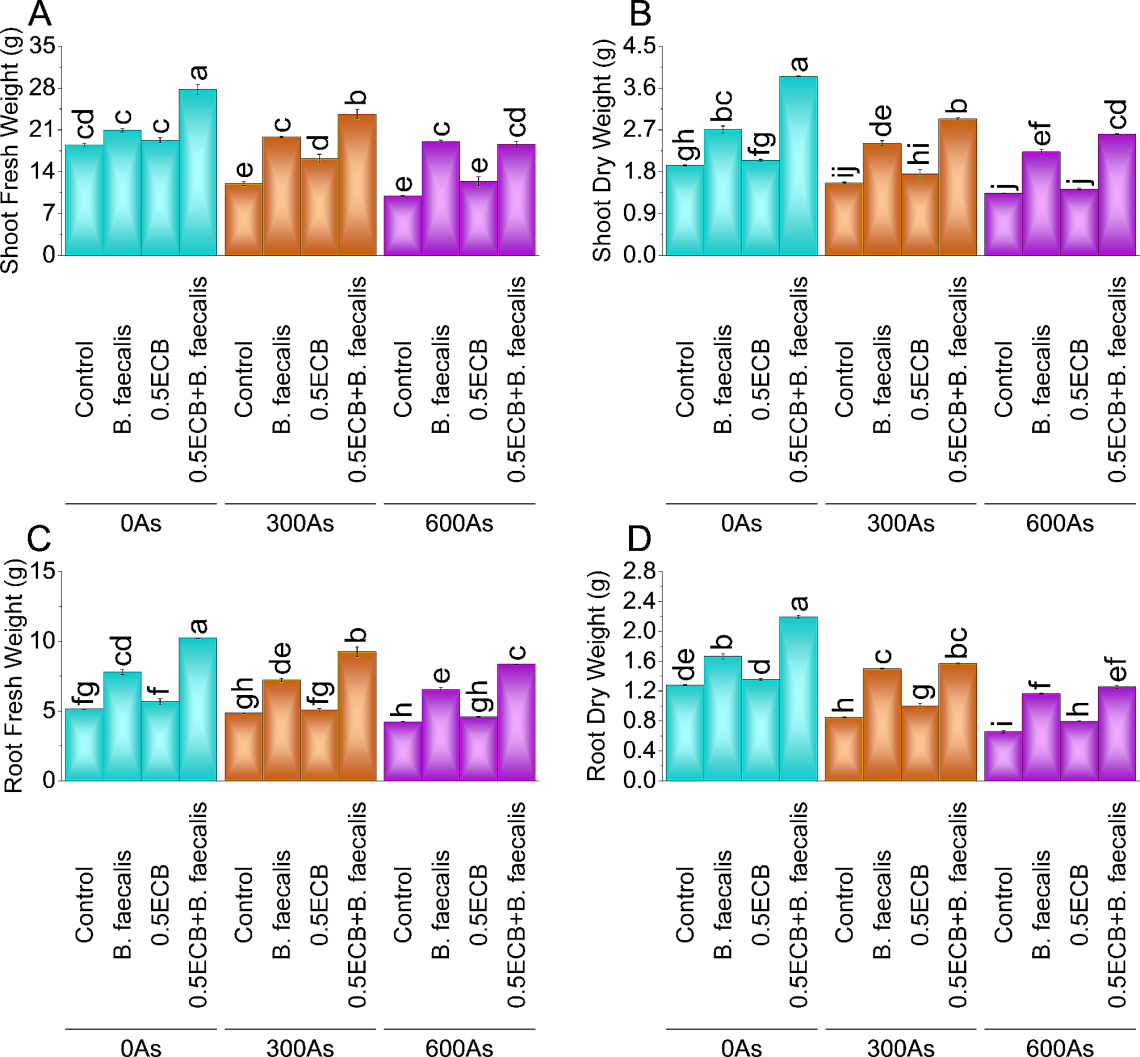
Influence of *B. faecalis* and composted biochar sole and combined application on maize shoot fresh weight (**A**), shoot dry weight (**B**), root fresh weight (**C**), and root dry weight (**D**) under 0 (0As), 300 (300As) and 600 mg As/kg soil (600As). Bars are an average of four replicates ± SE compared using Tukey’s test. Different letters showed significant changes at *p* ≤ 0.05

Under 0As stress, *B. faecalis* increased shoot fresh weight by ~ 13%, while 0.5ECB showed ~ 4% increase from the control. 0.5ECB + *B. faecalis* had the highest gain at ~ 50%. In 300As stress, *B. faecalis*, 0.5ECB, and 0.5ECB + *B. faecalis* increased by ~ 65%, ~ 35%, and ~ 97%, respectively, over control. For 600As stress, *B. faecalis*, 0.5ECB, and 0.5ECB + *B. faecalis* showed remarkable increases of ~ 93%, ~ 25%, and ~ 87%, respectively, over the control (Fig. 3A).

### Shoot dry weight

Compared to the control, *B. faecalis* significantly increased shoot dry weight by ~ 40%, ~ 55%, and ~ 67% under 0As, 300As, and 600As stress conditions, respectively. Similarly, 0.5ECB alone positively affected shoot dry weight, with percentage increases of ~ 6%, ~ 12%, and ~ 7% for the stress conditions. Notably the application of 0.5ECB + *B. faecalis* showed the highest percentage increase in shoot dry weight, recording values of ~ 99%, ~ 89%, and ~ 96% for the 0As, 300As, and 600As stress, respectively, over control (Fig. 3B).

### Root fresh weight

Under 0As stress, *B. faecalis* increased root fresh weight by ~ 52% and 0.5ECB by ~ 10%. The application of 0.5ECB + *B. faecalis* showed a remarkable ~ 99% elevation compared to the control. In the 300As stress condition, *B. faecalis* increased by ~ 49%, 0.5ECB by ~ 5% and the application of 0.5ECB + *B. faecalis* by ~ 91% over the control. For 600As, *B. faecalis* rose by ~ 55%, 0.5ECB by ~ 8%, and 0.5ECB + *B. faecalis* by ~ 97% compared to the control (Fig. 3C).

### Root dry weight

In the 0As stress condition, *B. faecalis* increased root dry weight by ~ 30%, and 0.5ECB by ~ 6%. The application of 0.5ECB + *B. faecalis* showed a remarkable ~ 71% elevation compared to the control. For 300As, *B. faecalis* increased by ~ 77%, 0.5ECB by ~ 18%, and 0.5ECB + *B. faecalis* by ~ 85% over the control. In the 600As stress condition, *B. faecalis* rose by ~ 78%, 0.5ECB by ~ 21%, and 0.5ECB + *B. faecalis* by ~ 91% compared to the control (Fig. 3D).

### Electrolyte leakage

**Fig. 4:**
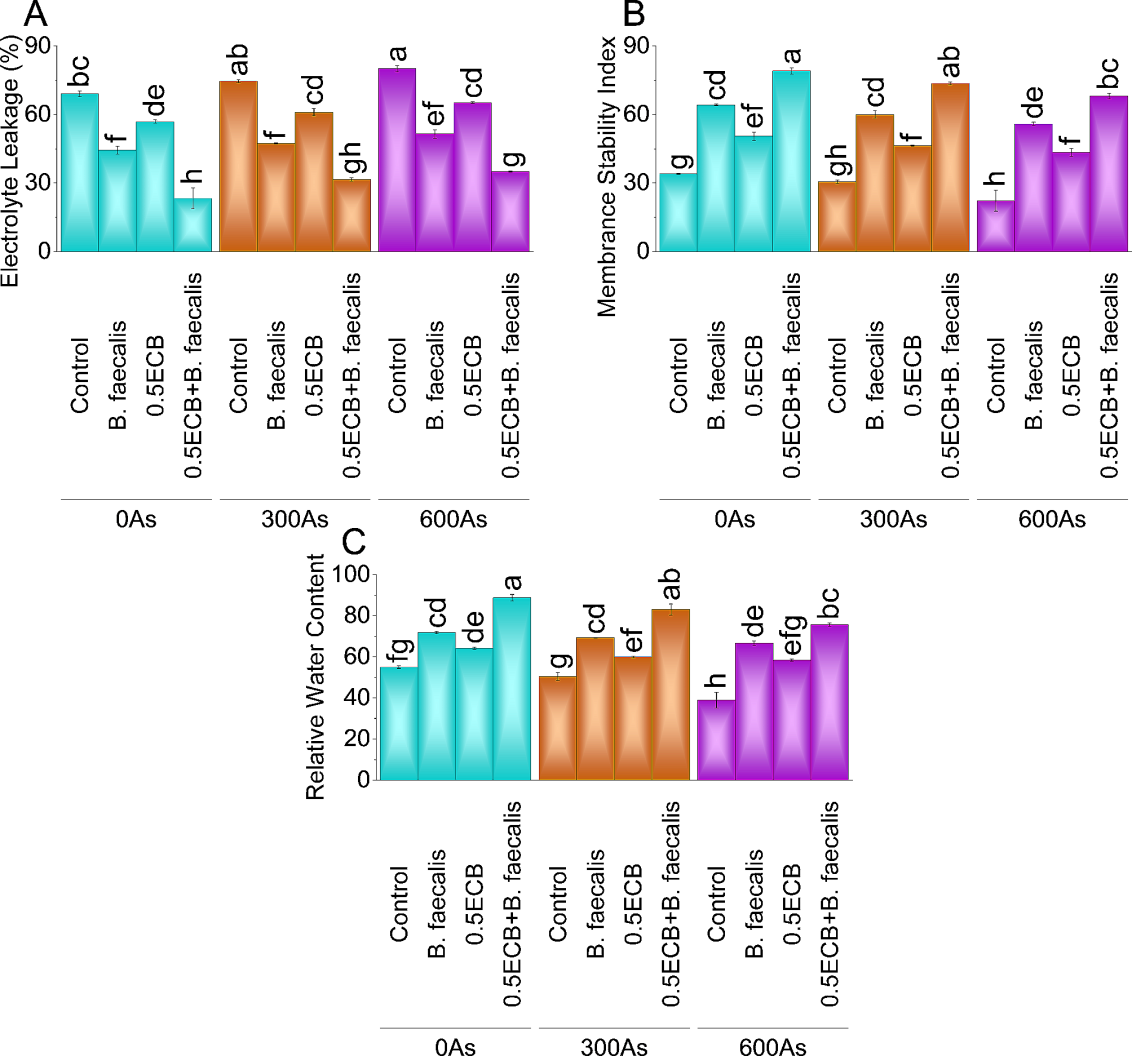
Influence of *B. faecalis* and composted biochar sole and combined application on maize electrolyte leakage (**A**), membrane stability index (**B**), and relative water content (**C**) under 0 (0As), 300 (300As) and 600 mg As/kg soil (600As). Bars are an average of four replicates ± SE compared using Tukey’s test. Different letters showed significant changes at *p* ≤ 0.05

In the 0As stress condition, *B. faecalis* reduced electrolyte leakage by ~ 36%, 0.5ECB by ~ 18%, and 0.5ECB + *B. faecalis* by ~ 66% compared to the control. Under 300As stress, *B. faecalis* decreased by ~ 37%, 0.5ECB by ~ 18%, and the application of 0.5ECB + *B. faecalis* by ~ 58%. In the 600As stress, *B. faecalis*, 0.5ECB, and 0.5ECB + *B. faecalis* decreased electrolyte leakage by ~ 36%, ~ 19%, and ~ 56%, respectively, compared to the control group (Fig. 4A).

### Membrane stability index (MSI)

Under 0As stress, *B. faecalis* increased MSI by ~ 89%, 0.5ECB by ~ 48%, and 0.5ECB + *B. faecalis* by ~ 132% over control. At 300As stress, *B. faecalis* increased MSI by ~ 96%, 0.5ECB by ~ 51%, and 0.5ECB + *B. faecalis* by ~ 140% in contrast to control. In the 600As stress condition, compared to the control, *B. faecalis*, 0.5ECB, and 0.5ECB + *B. faecalis* increased MSI by ~ 151%, ~ 95%, and ~ 206%, respectively (Fig. 4B).

### Relative water content

In the 0As stress condition, *B. faecalis* increased relative water content by ~ 31%, 0.5ECB by ~ 17%, and 0.5ECB + *B. faecalis* by ~ 61% compared to the control. Under 300As, *B. faecalis* increased by ~ 37%, 0.5ECB by ~ 19%, and the application of 0.5ECB + *B. faecalis* by ~ 64% over control. In the 600As stress, *B. faecalis*, 0.5ECB, and 0.5ECB + *B. faecalis* increased relative water content by ~ 71%, ~ 50%, and ~ 94%, respectively, compared to the control group (Fig. 4C).

### Antioxidants

Under 0As stress, *B. faecalis* decreased POD activity by ~ 15%, SOD activity by ~ 24%, and CAT activity by ~ 12% compared to the control. For 0.5ECB, the decrease was ~ 8% for POD activity, ~ 11% for SOD activity, and ~ 5% for CAT activity compared to the control under 0As stress. The combination treatment 0.5ECB + *B. faecalis* showed a decrease of ~ 23% for POD activity, ~ 34% for SOD activity, and ~ 20% for CAT activity over the control in 0As stress. At 300As stress, *B. faecalis* decreased POD activity by ~ 20%, SOD activity by ~ 22%, and CAT activity by ~ 13% compared to the control. For 0.5ECB, the decrease was ~ 9% for POD activity, ~ 9% for SOD activity, and ~ 8% for CAT activity from the control in 300As stress. The combination treatment 0.5ECB + *B. faecalis* showed a decrease of ~ 34% for POD activity, ~ 37% for SOD activity, and ~ 21% for CAT activity over the 300AS stressed control. In the 600As stress, *B. faecalis* decreased POD activity by ~ 7%, SOD activity by ~ 9%, and CAT activity by ~ 10% over the control. For 0.5ECB under 600As, resulted in ~ 2% decrease in POD activity, ~ 4% in SOD activity, and ~ 4% in CAT activity compared to the control. The combination treatment is 0.5ECB + *B. faecalis*, showed a decrease of ~ 14% for POD activity, ~ 15% for SOD activity, and ~ 15% for CAT activity from the control (Table 3).

**Table 3:** Influence of *B. faecalis* and composted biochar sole and combined application on maize peroxidase (POD), superoxide dismutase (SOD), catalase (CAT), ascorbate peroxidase (APX), hydrogen peroxide (H_2_O_2_), and malondialdehyde (MDA) under 0 (0As), 300 (300As) and 600 mg As/kg soil (600As)

**Treatment**	**POD** **(U/mg Protein)**	**SOD** **(U/mg Protein)**	**CAT** **(U/mg Protein)**
**0As**			
Control	24.56 ± 0.38 h	14.26 ± 0.24gh	51.42 ± 0.54 g
*B. faecalis*	21.44 ± 0.29ij	11.49 ± 0.20ij	45.86 ± 0.73hi
0.5ECB	22.75 ± 0.19hi	12.90 ± 0.28hi	48.83 ± 0.52gh
0.5ECB + *B. faecalis*	19.98 ± 0.21j	10.67 ± 0.17j	42.77 ± 0.34i
**300As**			
Control	36.81 ± 0.60d	21.51 ± 0.62d	66.52 ± 0.48d
*B. faecalis*	30.71 ± 0.49f	17.64 ± 0.51f	58.75 ± 0.14e
0.5ECB	33.64 ± 0.61e	19.69 ± 0.24e	61.68 ± 0.86e
0.5ECB + *B. faecalis*	27.54 ± 0.39 g	15.70 ± 0.17 g	55.08 ± 0.55f
**600As**			
Control	44.10 ± 0.05a	27.00 ± 0.22a	81.28 ± 0.74a
*B. faecalis*	41.16 ± 0.35b	24.72 ± 0.29bc	73.71 ± 0.82c
0.5ECB	43.09 ± 0.50ab	25.98 ± 0.14ab	77.84 ± 0.72b
0.5ECB + *B. faecalis*	38.82 ± 0.17c	23.38 ± 0.27c	70.62 ± 0.82c
**Treatment**	**APX** **(U/mg Protein)**	**H**_**2**_ **O**_**2**_ **(n mol/g FW)**	**MDA** **(nmol/mg Protein)**
**0AS**			
Control	2.22 ± 0.11 g	25.65 ± 0.60 g	0.58 ± 0.01hi
*B. faecalis*	1.77 ± 0.06hi	19.22 ± 0.32hi	0.39 ± 0.03jk
0.5ECB	1.95 ± 0.03gh	21.68 ± 0.55 h	0.48 ± 0.02ij
0.5ECB + *B. faecalis*	1.49 ± 0.13i	17.18 ± 0.36i	0.30 ± 0.02k
**300As**			
Control	3.56 ± 0.06d	41.23 ± 0.92d	0.95 ± 0.01de
*B. faecalis*	2.90 ± 0.02ef	33.06 ± 0.73f	0.77 ± 0.03 fg
0.5ECB	3.18 ± 0.04e	37.24 ± 0.70e	0.86 ± 0.03ef
0.5ECB + *B. faecalis*	2.58 ± 0.09f	29.22 ± 0.86 g	0.68 ± 0.03gh
**600As**			
Control	4.45 ± 0.06a	56.33 ± 0.90a	1.31 ± 0.01a
*B. faecalis*	4.02 ± 0.00bc	48.65 ± 0.90c	1.12 ± 0.01bc
0.5ECB	4.17 ± 0.06ab	52.30 ± 0.50b	1.20 ± 0.03ab
0.5ECB + *B. faecalis*	3.81 ± 0.04 cd	45.56 ± 0.83c	1.04 ± 0.03 cd

Values are an average of four replicates compared by using Tukey’s test. Different letters showed significant changes at *p* ≤ 0.05

Under 0As stress, *B. faecalis* exhibited a decrease in APX activity, H_2_O_2_, and MDA levels compared to the control, with decreases of approximately ~ 25%, ~ 33%, and ~ 48%, respectively. Similarly, the application of 0.5ECB decreased levels of APX activity, H_2_O_2_, and MDA by approximately ~ 14%, ~ 18%, and ~ 21%, respectively, from the control in 0AS. Moreover, the combined treatment of 0.5ECB and *B. faecalis* showed the most significant decrease in APX activity, H_2_O_2_, and MDA levels, with around ~ 49%, ~ 49%, and ~ 97%, respectively, over the control under 0AS. Under 300AS, *B. faecalis* exhibited a significant ~ 23%, ~ 25%, and ~ 24% decrease in APX activity, H_2_O_2_, and MDA levels, respectively. Likewise, 0.5ECB showed ~ 12%, ~ 11%, and ~ 10% reductions in APX activity, H_2_O_2_, and MDA levels, respectively, over the 300AS stressed control. The combined treatment of 0.5ECB and *B. faecalis* resulted in decreases of approximately ~ 38%, ~ 41%, and ~ 40% in APX activity, H_2_O_2_, and MDA levels, respectively, over the 300AS stressed control. In 600As stress, *B. faecalis* exhibited approximately ~ 11%, ~ 16%, and ~ 17% reductions in APX activity, H_2_O_2_, and MDA levels, respectively. Additionally, 0.5ECB demonstrated decreases of around ~ 7%, ~ 8%, and ~ 9% in APX activity, H_2_O_2_, and MDA levels, respectively, than the control under 600AS stress. The combined treatment of 0.5ECB and *B. faecalis* resulted in decreases of approximately ~ 17%, ~ 24%, and ~ 26% in APX activity, H_2_O_2_, and MDA levels, respectively, compared to the control (Table 3).

### Leaves nutrients concentration

**Fig. 5:**
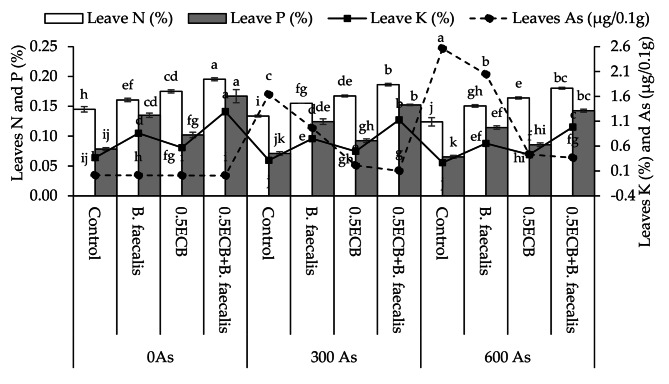
Influence of *B. faecalis*,* composted biochar sole*, and combined application on maize leaves N, P, K and As concentration under 0 (0As), 300 (300As) and 600 mg As/kg soil (600As). Bars are an average of four replicates ± SE compared using Tukey’s test. Different letters showed significant changes at *p* ≤ 0.05

Results showed that *B. faecalis*, 0.5ECB and 0.5ECB + *B. faecalis* caused significant enhancement of ~ 11, ~21 and ~ 35% respectively in leaves N over control at 0As. At 300As, ~ 16, ~25, and ~ 39% significant improvement was observed where *B. faecalis*, 0.5ECB and 0.5ECB + *B. faecalis* were applied respectively compared to control in leaves N. In case of 600As, *B. faecalis* showed ~ 22%, 0.5ECB resulted in ~ 32% and 0.5ECB + *B. faecalis* caused ~ 45% significant increase than control in leaves N (Fig. 5).

For leaves P, *B. faecalis*, 0.5ECB and 0.5ECB + *B. faecalis* showed significant increase of ~ 72, ~30 and ~ 113% respectively than control at 0As. In 300As, ~ 75, ~30, and ~ 115% significant enhancement was observed where *B. faecalis*, 0.5ECB and 0.5ECB + *B. faecalis* were applied respectively over control in leaves P. However, at 600As, *B. faecalis* showed ~ 75%, 0.5ECB resulted in ~ 31% and 0.5ECB + *B. faecalis* caused ~ 118% significant improvement from control in leaves P (Fig. 5).

Treatments *B. faecalis*, 0.5ECB and 0.5ECB + *B. faecalis* showed significant increase of ~ 133, ~55 and ~ 252% respectively than control at 0As in leaves K. In 300As, ~ 139, ~57, and ~ 252% significant increase was observed where *B. faecalis*, 0.5ECB and 0.5ECB + *B. faecalis* were applied respectively from control in leaves K. However, at 600As, *B. faecalis* showed ~ 146%, 0.5ECB resulted in ~ 61% and 0.5ECB + *B. faecalis* caused ~ 270% significant increment from control in leaves K.

Results showed that *B. faecalis*, 0.5ECB and 0.5ECB + *B. faecalis* caused decline of ~ 8, ~31 and ~ 46% respectively over control at 0As in leaves As. At 300As, ~ 41, ~87, and ~ 94% decline was observed where *B. faecalis*, 0.5ECB and 0.5ECB + *B. faecalis* were applied respectively compared to control in leaves As. However, at 600As, *B. faecalis* showed ~ 20%, 0.5ECB resulted in ~ 83% and 0.5ECB + *B. faecalis* caused ~ 86% decrease than control in leaves As (Fig. 5).

### Roots nutrients concentration

**Fig. 6:**
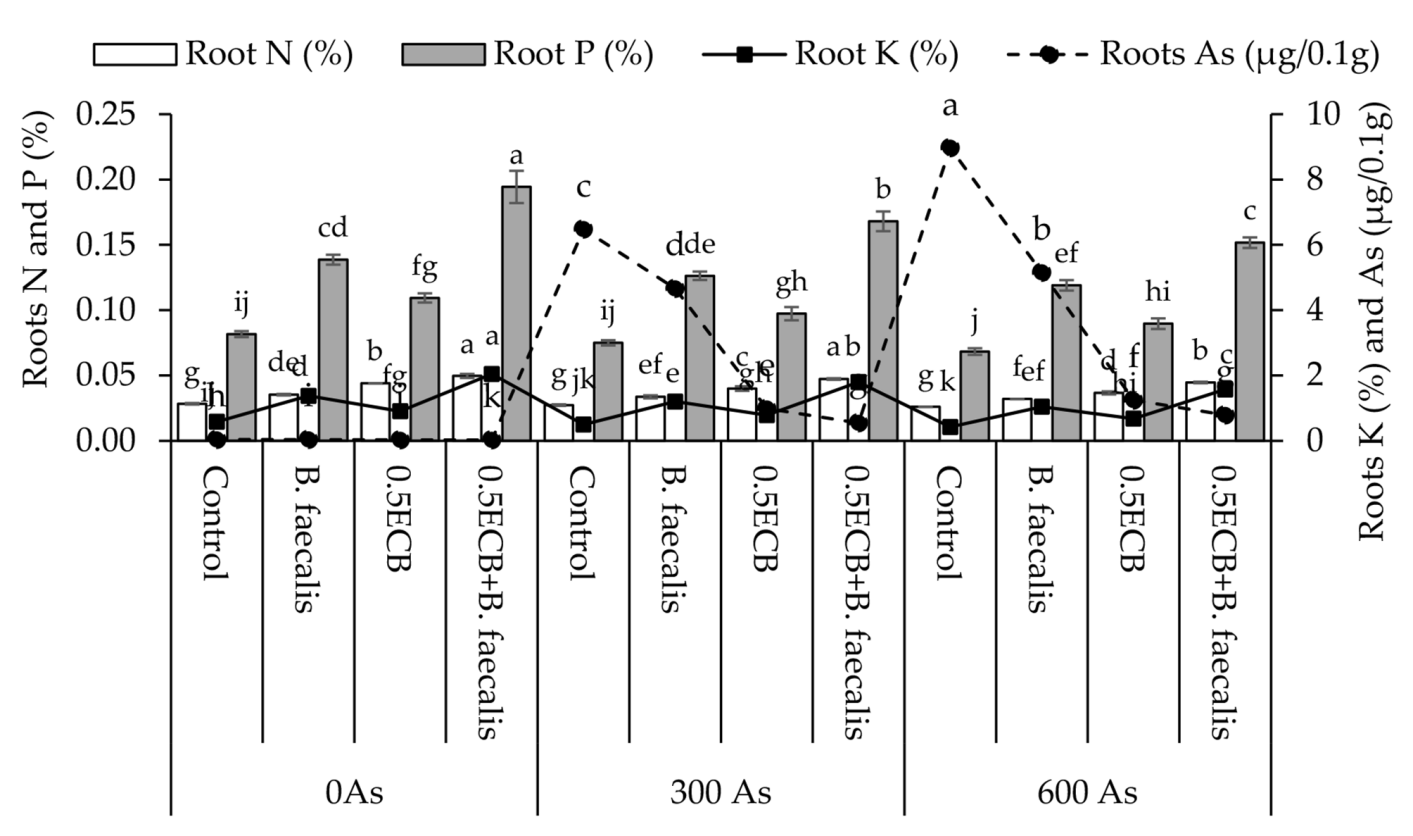
Influence of *B. faecalis* and composted biochar sole and combined application on maize roots N, P, K and As concentration under 0 (0As), 300 (300As) and 600 mg As/kg soil (600As). Bars are an average of four replicates ± SE compared using Tukey’s test. Different letters showed significant changes at *p* ≤ 0.05

Applying *B. faecalis*, 0.5ECB and 0.5ECB + *B. faecalis* caused significant improvement of ~ 25, ~55 and ~ 75% respectively in roots N over control at 0As. At 300As, ~ 23, ~46, and ~ 73% significant enhancement was observed where *B. faecalis*, 0.5ECB and 0.5ECB + *B. faecalis* were applied respectively compared to control in roots N. In case of 600As, *B. faecalis* showed ~ 23%, 0.5ECB resulted in ~ 41% and 0.5ECB + *B. faecalis* caused ~ 72% significant increments than control in leaves N (Fig. 6).

In the case of roots P, *B. faecalis*, 0.5ECB and 0.5ECB + *B. faecalis* showed significant increment of ~ 70, ~34 and ~ 138% respectively than control at 0As. In 300As, ~ 68, ~30, and ~ 124% significant improvement was observed where *B. faecalis*, 0.5ECB and 0.5ECB + *B. faecalis* were applied respectively over control in roots P. However, at 600As, *B. faecalis* showed ~ 74%, 0.5ECB resulted in ~ 31% and 0.5ECB + *B. faecalis* caused ~ 122% significant enhancement from control in roots P (Fig. 6).

Treatments *B. faecalis*, 0.5ECB and 0.5ECB + *B. faecalis* showed significant increase of ~ 70, ~34 and ~ 138% respectively than control at 0As in roots K. In 300As, ~ 68, ~30, and ~ 124% significant rise was observed where *B. faecalis*, 0.5ECB and 0.5ECB + *B. faecalis* were applied respectively from control in leaves K. However, at 600As, *B. faecalis* showed ~ 74%, 0.5ECB resulted in ~ 31% and 0.5ECB + *B. faecalis* caused ~ 122% significant increment from control in leaves K.

Under 0As, *B. faecalis*, 0.5ECB and 0.5ECB + *B. faecalis* showed significant decrease of ~ 7, ~32 and ~ 40% respectively than control at 0As in roots As. In 300As, ~ 28, ~85, and ~ 92% significant decline was observed where *B. faecalis*, 0.5ECB and 0.5ECB + *B. faecalis* were applied respectively from control in leaves As. However, at 600As, *B. faecalis* showed ~ 43%, 0.5ECB resulted in ~ 86% and 0.5ECB + *B. faecalis* caused ~ 91% significant decline from control in leaves As (Fig. 6).

### Chlorophyll contents

**Fig. 7:**
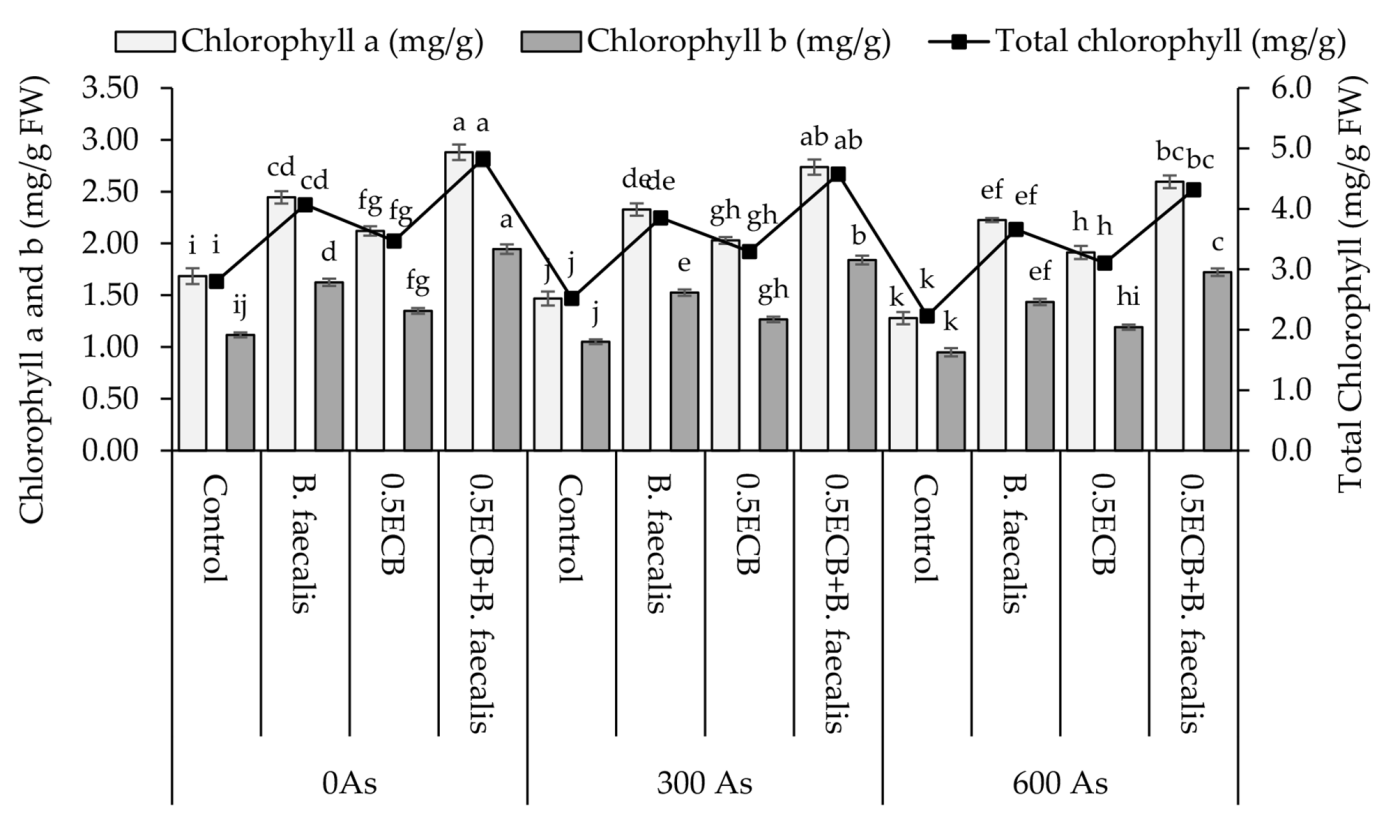
Influence of *B. faecalis* and composted biochar sole and combined application on maize leaves chlorophyll a, b and total contents under 0 (0As), 300 (300As) and 600 mg As/kg soil (600As). Bars are an average of four replicates ± SE compared by using Tukey’s test. Different letters showed significant changes at *p* ≤ 0.05

Applying *B. faecalis*, 0.5ECB and 0.5ECB + *B. faecalis* caused significant improvement of ~ 45, ~26 and ~ 71% respectively in chlorophyll a over control at 0As. At 300As, ~ 59, ~38 and ~ 86% significant enhancement was observed where *B. faecalis*, 0.5ECB and 0.5ECB + *B. faecalis* were applied respectively compared to control in chlorophyll a. In case of 600As, *B. faecalis* showed ~ 74%, 0.5ECB resulted in ~ 50% and 0.5ECB + *B. faecalis* caused ~ 99% significant increments than control in chlorophyll a (Fig. 7).

In the case of chlorophyll b P, *B. faecalis*, 0.5ECB and 0.5ECB + *B. faecalis* showed significant increment of ~ 45, ~21 and ~ 74% respectively than control at 0As. In 300As, ~ 45, ~20 and ~ 75% significant improvement was observed where *B. faecalis*, 0.5ECB and 0.5ECB + *B. faecalis* were applied respectively over control in chlorophyll b. However, at 600As, *B. faecalis* showed ~ 51%, 0.5ECB resulted in ~ 25% and 0.5ECB + *B. faecalis* caused ~ 81% significant enhancement from control in chlorophyll b (Fig. 7).

Treatments *B. faecalis*, 0.5ECB and 0.5ECB + *B. faecalis* showed significant increase of ~ 45, ~24 and ~ 72% respectively than control at 0As in total chlorophyll. In 300As, ~ 53, ~31, and ~ 82% significant rise was observed where *B. faecalis*, 0.5ECB and 0.5ECB + *B. faecalis* were applied respectively from control in total chlorophyll. However, at 600As, *B. faecalis* showed ~ 64%, 0.5ECB resulted in ~ 39% and 0.5ECB + *B. faecalis* caused a ~ 94% significant increment from control in total chlorophyll (Fig. 7).

## Discussion

Maize, a nutritious cereal grain, is rich in macronutrients, micronutrients, and essential mineral ions. It also contains antioxidants with therapeutic potential [15]. In addition to examining the potential of ECB and *B. faecalis* in reducing arsenic toxicity, this study looked at the effect of biochar on maize quality under arsenic stress. According to the results, the combination application of Bacillus faecalis and 0.5ECB significantly enhanced growth metrics and biomass, especially under severe arsenic stress, indicating a synergistic impact to enhance maize growth.

Important growth indices in maize crops, such as plant height, leaf length, leaf width, shoot length, and root length, are significantly impacted by arsenic pollution [65]. This disruption is due to arsenic’s interference with root development, nutrient absorption, and overall plant growth [66]. Similarly, biomass parameters such as shoot fresh weight, shoot dry weight, root fresh weight, and root dry weight experience significant reductions under arsenic-induced stress, as arsenic disrupts photosynthetic processes and diminishes biomass production [67]. This stress also affects physiological attributes like the number of leaves and leaf index, leading to compromised foliar development and altered leaf morphology. Arsenic stress further permeates cellular and physiological levels, evident in increased electrolyte leakage, decreased relative water content, and declining membrane stability index, indicating compromised membrane integrity and susceptibility to oxidative stress [68, 69]. These findings highlight the vulnerability of maize crops to arsenic-induced disturbances, potentially affecting overall plant health and resilience [70, 71].

Our study showed that 0.5ECB (composted biochar) had a significant positive impact on key maize growth factors under arsenic stress, effectively mitigating the harmful effects of arsenic contamination [72–76]. Applying 0.5ECB led to a notable decrease in arsenic absorption by maize roots and leaves. This result is attributed to 0.5ECB’s unique properties and interactions within the maize rhizosphere. Composted biochar is a beneficial soil amendment with diverse effects derived from organic matter through pyrolysis. In our research, the porous structure of 0.5ECB played a crucial role in reducing arsenic bioavailability in the soil, creating an environment that limits arsenic mobility and uptake by plant roots [77]. This finding is consistent with previous studies highlighting biochar’s ability to adsorb heavy metals and restrict their movement in the soil, thus decreasing their accessibility to plants [78, 79]. Composted biochar can significantly influence how plants absorb nutrients. Its porous structure acts like a sponge, holding onto essential elements such as nitrogen, phosphorus, and potassium, which would otherwise be lost through leaching [80]. This retention ensures these nutrients remain available to plants over time, promoting healthier growth [81]. Another beneficial aspect is its ability to enhance nutrient uptake remains efficient and safe. However, the specific impacts on phosphorus and potassium uptake can vary based on factors like biochar type, soil conditions, and plant species, necessitating careful consideration in agricultural applications [82].

Introducing 0.5ECB into the soil improved its structure and nutrient retention, creating a healthier soil environment for maize growth, consistent with prior research [83]. The observed enhancements in maize growth parameters under arsenic stress, such as plant height, leaf length, leaf width, shoot length, and root length, are attributed to 0.5ECB’s ability to immobilize arsenic and enhance soil health [84]. These findings highlight 0.5ECB’s potential for promoting plant resilience and mitigating arsenic contamination in agricultural soils. Similarly, the application of *Bacillus faecalis* (*B. faecalis*) positively impacted various parameters under arsenic stress in our maize crop study. *B. faecalis* mitigated the adverse effects of arsenic on plant growth parameters, including plant height, leaf length, leaf width, shoot length, root length, and biomass [85, 86]. This effectiveness may be due to *B. faecalis* role as a rhizobacterium, influencing nutrient availability, promoting root development, and regulating plant metal uptake, as supported by previous studies [87]. Comparing our findings with existing literature, *B. faecalis* demonstrates consistent potential in improving plant growth parameters and alleviating metal-induced toxicity in various crops [88, 89]. The combined use of 0.5ECB and *B. faecalis* effectively boosted maize crop parameters amidst arsenic stress. It notably enhanced plant height, leaf dimensions, and biomass attributes, indicating healthier growth. The intervention also reduced electrolyte leakage, signifying improved membrane stability and stress response. The study suggests that employing 0.5ECB and *B. faecalis* could be a sustainable strategy for enhancing maize resilience in arsenic-contaminated soils. Further research should delve into its real-world application and long-term effects to comprehensively understand its efficacy and scalability.

## Conclusions

In summary, using 0.5ECB combined with *Bacillus faecalis* (*B. faecalis*) can potentially enhance maize growth under arsenic-induced stress. This treatment showed potential in regulating antioxidants against arsenic, thus helping to reduce the harmful effects of arsenic stress on maize plants. Further extensive field studies are needed to confirm the effectiveness of 0.5ECB combined with *Bacillus faecalis* as a viable solution for alleviating arsenic-induced stress in maize plants.

## Data Availability

All data generated or analysed during this study are included in this published article.
